# New Perspectives for Evaluating the Mass Transport in Porous Catalysts and Unfolding Macro- and Microkinetics

**DOI:** 10.1007/s10562-022-04218-6

**Published:** 2022-12-07

**Authors:** Stefan Wild, Christoph Mahr, Andreas Rosenauer, Thomas Risse, Sergey Vasenkov, Marcus Bäumer

**Affiliations:** 1 Institute for Applied and Physical Chemistry, University of Bremen, 28359 Bremen, Germany; 2 MAPEX Center of Materials and Processes, University of Bremen, 28359 Bremen, Germany; 3 Institute of Solid State Physics, University of Bremen, Otto-Hahn-Allee 1, 28359 Bremen, Germany; 4 Institute of Chemistry and Biochemistry, Free University Berlin, 14195 Berlin, Germany; 5 Department of Chemical Engineering, University of Florida, Gainesville, FL 32611 USA

**Keywords:** Porous heterogeneous catalysts, Tortuosity factor, Diffusive mass transport, Catalyst effectiveness factors, Pulsed field gradient NMR, TEM tomography, Nanoporous gold

## Abstract

In this article we shed light on newly emerging perspectives to characterize and understand the interplay of diffusive mass transport and surface catalytic processes in pores of gas phase metal catalysts. As a case study, nanoporous gold, as an interesting example exhibiting a well-defined pore structure and a high activity for total and partial oxidation reactions is considered. PFG NMR (pulsed field gradient nuclear magnetic resonance) measurements allowed here for a quantitative evaluation of gas diffusivities within the material. STEM (scanning transmission electron microscopy) tomography furthermore provided additional insight into the structural details of the pore system, helping to judge which of its features are most decisive for slowing down mass transport. Based on the quantitative knowledge about the diffusion coefficients inside a porous catalyst, it becomes possible to disentangle mass transport contributions form the measured reaction kinetics and to determine the kinetic rate constant of the underlying catalytic surface reaction. In addition, predictions can be made for an improved effectiveness of the catalyst, i.e., optimized conversion rates. This approach will be discussed at the example of low-temperature CO oxidation, efficiently catalysed by npAu at 30 °C. The case study shall reveal that novel porous materials exhibiting well-defined micro- and mesoscopic features and sufficient catalytic activity, in combination with modern techniques to evaluate diffusive transport, offer interesting new opportunities for an integral understanding of catalytic processes.

**Graphical Abstract:**

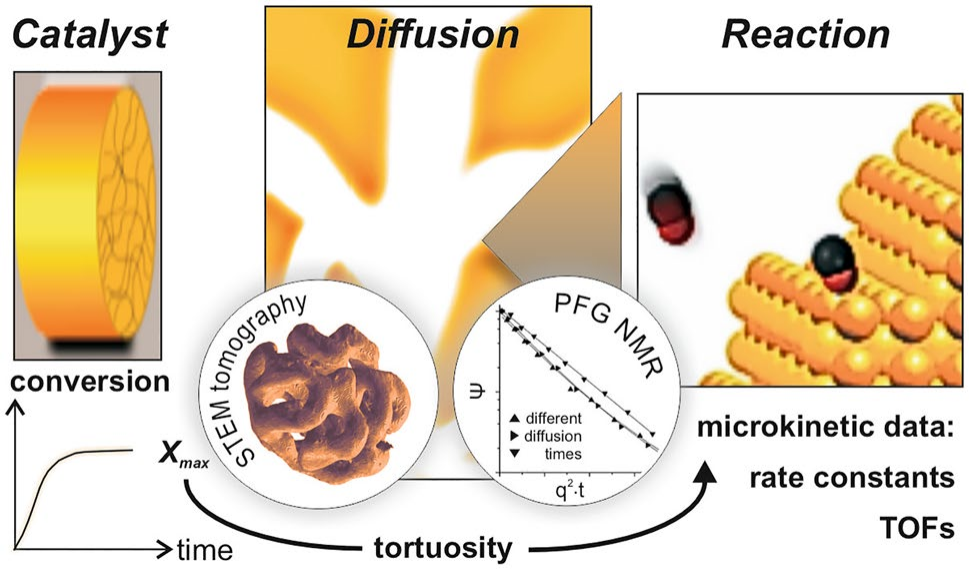

## Introduction

**Fig. 1:**
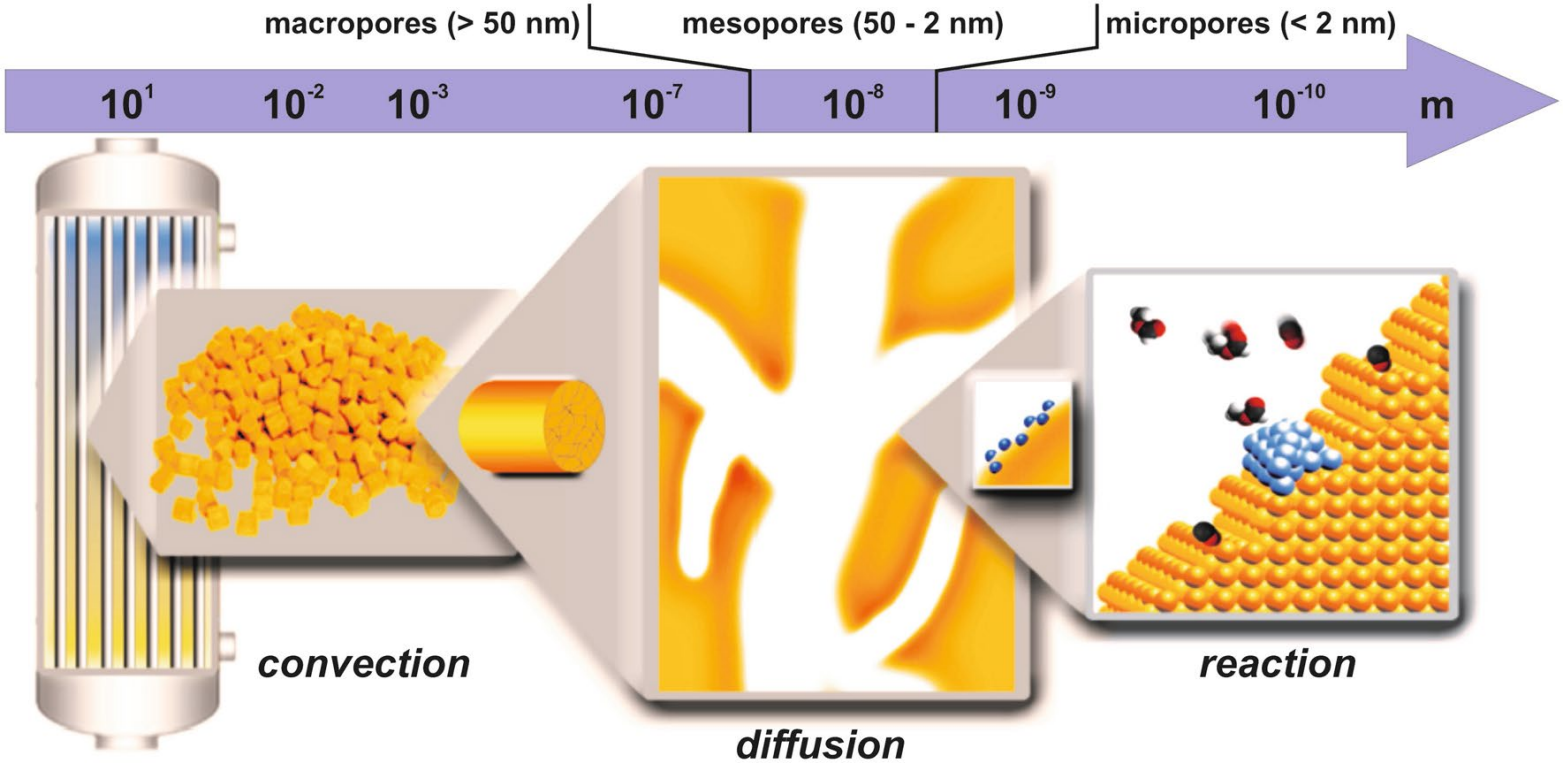
Schematic sketch of the various length scales which are important for catalytic processes; diffusive transport can be of relevance in the range between a few 100 nm and a few nm. In particular, within the pore network of porous catalysts mass transport is only accomplished in this way. Pores are classified into micropores (single molecule or confined diffusion), mesopores (Knudsen and molecular diffusion) and macropores (typically dominated by molecular diffusion), according to their diameter

In heterogeneous catalysis, a general problem arises from two conflicting demands. On the one hand, large surface areas are necessary to achieve sufficient conversion levels. Therefore, porous materials are used—typically as supports for finely-dispersed nanoparticulate catalysts but also in form of porous bulk catalysts [1]. On the other hand, however, high specific surface areas are affiliated with small pore diameters which, in turn, can cause mass transport limitations [2]. These can severely reduce the overall catalyst performance, when the diffusion of reactants and products to/from the catalytically active areas or sites on the catalyst’s surface is slower than the actual surface reaction. A catalytic process, as operated in industry, for instance, is schematically sketched in Fig. 1. While on the reactor scale convection dominates and can be adjusted and optimized, at small scales diffusion is the only transport mechanism for reactants to access the surfaces of micropores and small mesopores [2]. In contrast to convection, the diffusive mass transport in such pores cannot be easily quantified even if the process conditions are well known. The diffusive mass transport depends on structural factors of the catalyst, pore wall-gas interactions, and the resulting diffusivities of the involved gases [2].

Apart from film diffusion at the outer surface of a catalyst particle, diffusion within the pore system is a major factor that affects mass transport and can limit the productivity [1]. On the one hand, Knudsen diffusion which is slower than diffusion in the bulk gas phase can contribute to this effect. It occurs when the pore diameters are smaller than the mean free path of the molecules so that collisions of gas molecules with the pore walls prevail over intermolecular collisions. On the other hand, the structural constitution of the pore system, being characteristic for a specific catalyst material, can elongate the diffusive pathways and slow down the mass transport depending on the shape of the pores, their connectivity and pore size distribution [2].

Typically, the majority of porous catalysts exhibit mesopores in a range of a few 10 nm (see, Fig. 1). They ascertain a sufficiently large specific surface area while, at the same time, usually not posing too high diffusion resistances for the reaction gases. In cases where the catalyst preparation method allows deliberately introducing a certain number of additional macropores, hierarchically organized pore systems may be created, in which the latter take the role of “gateways” and act as distributors for the mesopore ensemble [3]. Micropores, hampering diffusion most but increasing the specific surface area substantially, occur material specific [4]. Zeolites, for instance, comprise them due to their crystallography [4], while in case of activated carbon [5] they are the result of the preparation process.

Yet, such a classification is not sufficient to estimate how severely diffusion limitations will degrade a catalyst’s performance. To assess such impediments, as given by the specific type and the structural characteristics of a pore system, a quantity, called tortuosity τ, was introduced which integrally quantifies to which degree diffusion in porous solids is slowed down in comparison to the bulk gas phase (*τ* > 1) [2]. A structurally more differentiated perspective takes into account that pathway elongations (due to non-straight, but tortuous pores) as well as strongly varying pore diameters or pore cross sections, respectively, can play a role. The latter aspect comes into play when locations in the pore system, where the pore diameters are minimal, represent “bottle necks” for the diffusing molecules which they have to overcome. On this basis, two parameters are needed to characterize the pore system: one, also called tortuosity in the literature, and another one, called constrictivity (*δ *<1) [2].

Understanding and modelling transport processes in porous catalysts is an important topic under several perspectives, but not as much in the focus of fundamental research of the catalytic community as compared to questions regarding the catalytic surface reactions, i.e., the catalytic properties of a material. In practical applications, however, lower product yields caused by transport limitations will cut profits [6]. Accordingly, in corporate R&D (research and development) significant effort is undertaken to develop catalyst formulations that guarantee facile accessibility of the catalytically active areas or sites for the reactants and an optimized removal of the products.

But also in scientific studies, a quantitative consideration of mass transport contributions to the results of catalytic experiments is inevitable when, for instance, evaluating the catalytic properties of a novel material. Unless transport limitations can be avoided experimentally [7–9], measured conversions do not necessarily reflect the intrinsic catalytic activity, i.e., the maximum number of chemical turnovers which could (in principle) take place on a catalyst’s surface. In case the supply of reactants by diffusion is not fast enough, the apparent (observable) kinetics, also dubbed macrokinetics, will not represent the kinetics of the underlying catalytic surface reactions, called microkinetics in the following [10]. Aiming at characterizing and quantifying the latter, data about the diffusion within the catalyst is needed to be able to extract this information from the experimental results [11].

In 1939, Ernest W. Thiele proposed a concept which allows quantitatively assessing the influence of mass transport limitations on heterogeneously catalyzed reactions [12]. To this end, he introduced a dimensionless number, later called Thiele modulus, which unites all relevant quantities. Apart from a length characterizing the macroscopic geometry of the catalyst particles used and reflecting the relevant dimension for the diffusion profiles, it contains the ratio of the microkinetic rate constant and the effective diffusion coefficient within the porous material [1]. Using this well-established concept, it is possible not only to calculate the reduction of the catalyst’s effectiveness due to mass transport limitations in a straightforward and elegant fashion, but also to optimize transport properties and, in turn, the yield of products based on predictions or simulations, respectively [13].

A problem, however, often existing in this context relates to missing knowledge about those porosity-related features of a material, which determine gas diffusivities within its pore system. Basically, two experimental options are available to get such information: (i) STEM (scanning transmission electron microscopy) tomography [14–17] as well as related techniques and, (ii) techniques capable of microscopic diffusion measurements, such as pulsed field gradient nuclear magnetic resonance (PFG-NMR [2, 18]). While (i) is based on 3D reconstructions of the mesoscopic pore structure, from which these quantities can be numerically derived or estimated, (ii) allows to measure the diffusion of gas molecules within the pores directly. Over the last years, both methodologies have revealed an outstanding potential and helped to lay the foundation for a quantitative understanding of mass transport processes in porous matter, as will become obvious in this perspective article.

Particularly interesting in this context are materials with well-defined and homogeneous pore structures, guaranteeing identical or similar diffusion throughout a catalyst particle. Since modern materials science, and especially the realm of nanotechnological structuring options has borne a wealth of novel approaches to prepare porous catalysts with tailored properties, such materials become increasingly available [19]. In addition, if the catalytic surface properties do not change at different locations within the pore system, fascinating new opportunities result that allow maximizing catalytic activity and minimizing transport limitations, i.e., optimizing the productivity of a catalyst.

To illustrate this aspect, we will revert in this article to nanoporous gold (npAu) as a case study [20]. This novel skeletal metal catalyst has attracted considerable attention over the last years [21–25], after its high catalytic activity for total as well as partial oxidation reactions at low temperatures was discovered in 2006 and 2010, respectively [26–28]. Exhibiting a well-defined and tunable mesoporosity, npAu is particularly well-suited to disentangle the microkinetics and the mass transport properties if reactivity measurements are combined with techniques to quantify diffusion properties, i.e., PFG NMR and (S) TEM tomography.

After briefly introducing npAu and its structural characteristics, we will shed light on the following topics—each presented in an own section of this article:

- Dependence of the specific surface area on the mean ligament size in case of well-ordered pore structures
- Diffusion of gases and gas mixtures in porous matter
- Direct measurement of gas diffusivities in porous materials by PFG NMR
- Determination of tortuosities by STEM tomography
- Relationship between macro- and microkinetics
- Optimization of a catalyst’s performance by reducing mass transport limitations

## Nanoporous Gold as a Prototype of a Well-Defined Porous Metal Catalyst

Although in heterogeneous catalysis the main class of porous materials applied are oxides (used as supports for noble metal catalysts e.g.), also porous metals belong to the portfolio of industrially employed catalysts [1]. A prominent example of that kind is Raney nickel, which is synthesized by leaching Al out of a NiAl alloy under alkaline conditions [29]. As a result, a powder is obtained consisting of porous Ni particles. Nanoporous gold is actually prepared in a similar way, namely by leaching a less noble metal out of an Au containing alloy, such as AuAg. In contrast to Raney nickel, however, the process is carried out in an acid. The corrosion process, often called dealloying, can be performed with or without control of the electrochemical potential, utilizing a potentio- or galvanostatic setup [30, 31]. Under purely chemical conditions, the process is referred to as free corrosion and is particularly easy to carry out practically. Upon submersing an AuAg alloy specimen containing between 60 and 80 at% Ag—for instance in form of a thin disc (several mm in diameter and a few 100 microns in thickness)—in conc. HNO_3_, almost all Ag is leached out of the material over a time period of 12–24 h (resulting in residual Ag bulk contents of typically ≤ 1 at% [30]).

**Fig. 2:**
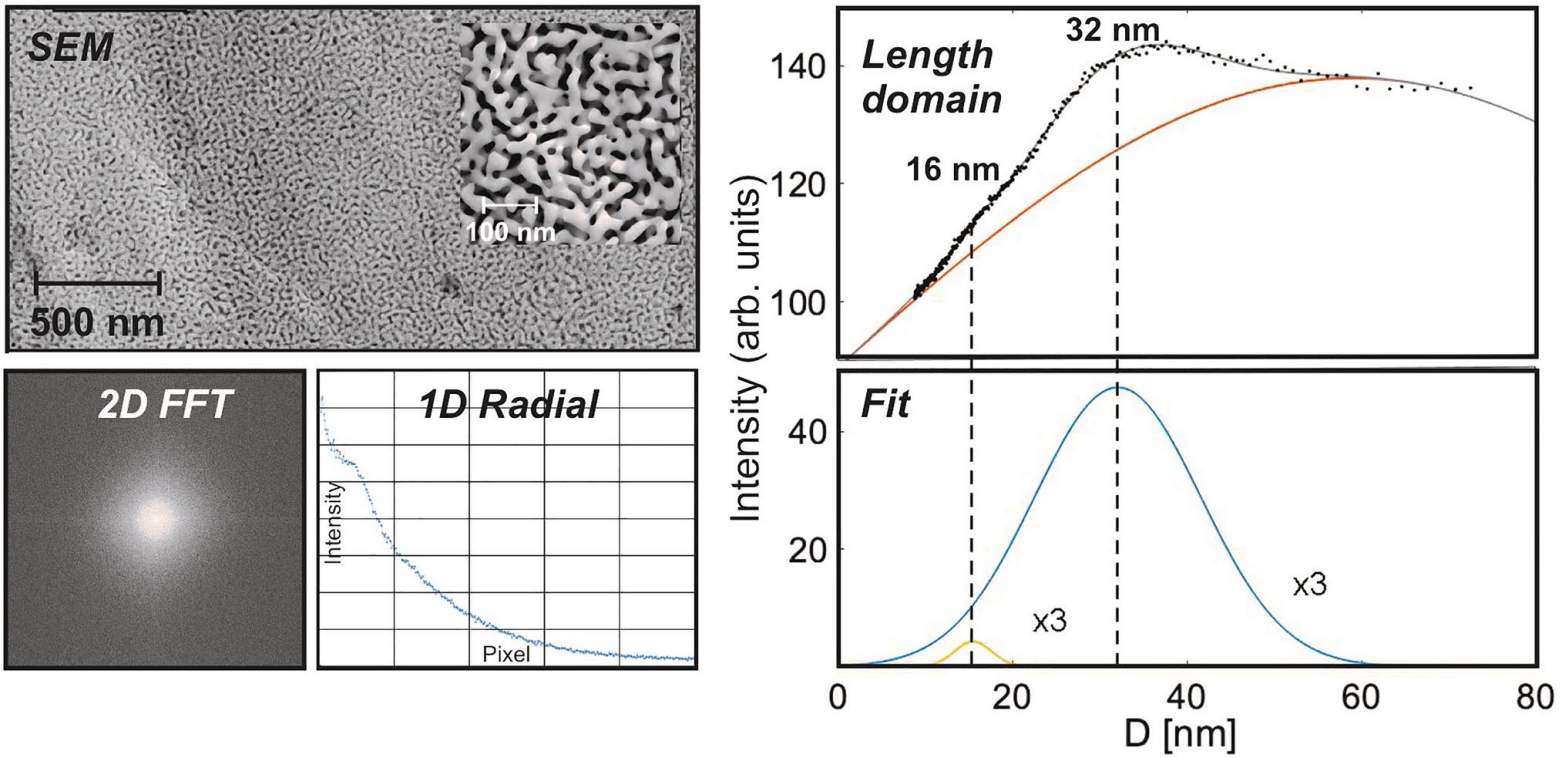
Structural characterization of npAu (prepared by free corrosion) and evaluation of mean pore and ligament sizes: To this end, the SEM micrograph (top left) is Fourier transformed. The 2D (spatial) frequency domain reveals the isotropy of the pore structure (bottom left) and can be radially integrated (bottom middle). Converted back into the length domain (top right) and fitted (bottom right), two maxima can be discerned—in addition to a broad background. These can be assigned to the mean value of the distance from ligament to ligament (i.e., the sum of ligament and pore diameter) in case of the pronounced maximum (at 32 nm) and to the individual values of pore and ligament size, respectively, in case of the small maximum (at 16 nm), indicating that the latter are identical. (In contrast, the broad background, exhibiting a maximum around 60 nm, is likely to originate from periodicities perpendicular to the pore/ligament diameters, i.e., is related to the ligament lengths.)

During the process, where Ag is continuously removed from the solid and dissolved as Ag^+^(aq), Au surface atoms become mobile and diffuse from and to step edges [32, 33]. In this way, large structural rearrangements are evoked which are accompanied by the concomitant formation of void spaces, gradually resulting in extended pores [32, 33]. When the dealloying process finally comes to an end, a highly porous bi-continuous nanostructure is formed, comprising ligaments and pores in the mesoporous regime (5–50 nm). In contrast to Raney nickel, the material does not break up into small particles during the synthesis so that monoliths are obtained, exhibiting the shape and grain structure of the original alloy specimen [34]. The pore systems found in these monoliths are structurally homogenous, as can be inferred from Fig. 2, showing an exemplary SEM micrograph acquired at the outer surface of a disc-shaped sample. Typically, the same porosity is observed throughout the material, i.e., also in the interior of such a npAu disc [35].

The ring-shaped feature in the 2D Fourier transformation of the SEM picture (see also ref. [36] in this context), which is also depicted in Fig. 2 (2D FFT, bottom left), indicates that the structure is spatially isotropic. After radially integrating the 2D FFT image (Fig. 2, bottom middle) and converting the x-axis from reciprocal values to distances in real space again (Fig. 2, top right), the resulting curve reveals periodically occurring lengths. In case of the example presented in Fig. 2, two maxima are discernible. The more pronounced one at 32 nm is ascribable to the mean value of ligament plus pore sizes, which is the prominent distance occurring in the pore structure. The less intense maximum corresponds to half of this value (16 nm). Based on the observation that the average ligament and pore diameters are typically identical for npAu [37, 38], it can be assigned to the individual values, i.e., the mean pore and ligament size.

For as-prepared samples (free corrosion), typically ligament sizes between 20 and 50 nm are obtained [30, 31]. It is worth noting, however, that the initial ligament diameter can also be increased (up to several 100 nm) by subsequently annealing the material in a controlled fashion [39]. Interestingly and characteristic of npAu, the thermally induced coarsening takes place without a change of the shape and the connectivity of ligaments and pores [39]. In this way, npAu samples with differing mean pore sizes but self-similar pore systems can be generated [37, 40–42], illustrating what has been alluded to already in the Introduction—namely options to tailor catalytic materials based on knowledge from materials science.

As will be shown in the following sections, catalysts, such as npAu, which can be prepared with a well-defined and spatially homogeneous pore structure ensure predictable surface areas and conditions for diffusive transport. Furthermore, identical or similar surface and thus catalytic properties can be expected under these circumstances throughout the material. This perspective article aims at elucidating that and how such features lay the foundation for a meaningful disentanglement of macro- and microkinetics as well as for an understanding and description of the catalytic process as a whole [43].

NpAu has been chosen as a case study in this context because of its ability to efficiently catalyze total oxidations as well as partial oxidations under aerobic conditions. The first studies reporting high activity for CO oxidation at room temperature and even below attracted attention as this observation was unexpected—taking into account that supported Au nanoparticles, which have been studied already since the 1980’s[44], show activity only in a very narrow range of sizes (a few nm), being an order of magnitude smaller than npAu’s ligaments. Without going into details here—the interested reader is referred to a number of extensive and excellent reviews on this topic [20, 22, 23, 25]—npAu’s catalytic potential arises from a combination of different features. On one hand, it comprises a high density of low-coordinated surface atoms making up as much as a fifth (20%) of all surface atoms [45]. For CO as an adsorbate, for instance, it has been shown that it can bind there much more strongly than on regular Au sites [46, 47]. Adsorption energies between 0.4 and 0.6 eV were experimentally determined and theoretically predicted [46], as compared to a value of 0.3 eV which was calculated for an ideal Au (111) surface [48]. On the other hand, residual silver remaining in the material after dealloying is found at the surface in concentrations which are up to an order of magnitude larger than in the bulk of the ligaments [49, 50]. Various theoretical studies provided ample evidence that Ag plays an important part for the oxidation reactions taking place on the surface of npAu (see e.g. ref. [51]). Since binding of molecular oxygen on Au is too weak to allow for its dissociative adsorption, it was concluded that Ag incurs the role of an oxygen activating species. Even though it has not been finally clarified whether metallic silver areas, bimetallic ensembles or oxidic Ag species are responsible, it seems clear that the two metals interact synergistically in the catalytic cycles on the surface.

Within this perspective article, we will focus on low-temperature CO oxidation, since former studies revealed a comparatively simple reaction kinetics in this case which facilitates the following discussion. Specifically, a reaction order of ~ 1 was found for CO and one close to zero for oxygen [24, 52], suggesting that the supply of oxygen on the surface is distinctly faster than the supply of CO so that the former is kinetically not relevant. Since the Thiele formalism predicts that a reaction order of 1 does not change under mass transport limiting conditions (for other reaction orders this is not the case), for both, the macro- (*r*_*macro*_) and the microkinetics (*r*_*micro*_), a rate law of 1st order can be assumed which just depends on the partial pressure of CO (*p(CO)*):

1 $$r_{micro} = k_{V} \cdot p\left( {CO} \right) = k_{A} \cdot A_{V} \cdot p\left( {CO} \right)$$

$$k_{V} :\,rate{\ }constant{\ }related{\ }to{\ }volume,{\ }k_{A} :rate{\ }constant{\ }rel.{\ }to{\ }catalytic{\ }surface{\ }area{\ }A,$$ $$A_{V} : specific{\ }surface{\ }area{\ }per{\ }volume,{\ }p\left( {CO} \right):CO{\ }partial{\ }pressure$$

In the context of heterogeneously catalyzed reactions, it has to be taken into account that, while in standard kinetics rate laws are typically based on volume-related rate constants *k*_*V*_ (mol/(m^3^⋅s)), the use of rate constants related to the surface area of the catalyst *A* is more appropriate in the former case (*k*_*A*_ (mol/(m^2^⋅s), see Eq. 1). Both are connected by the specific surface area *A*_*V*_ per volume catalyst (m^2^/m^3^ = 1/m; see also next section).

## Dependence of the Specific Surface Area on the Ligament Size in Case of Well-Ordered Pore Structures

The specific surface area *A*_*m*_ per mass (m^2^/g) is an important characteristic feature of a porous catalyst. Experimental techniques to determine it are physisorption measurements (with N_2_ or Ar) or cyclic voltammetry (CV). Both of them, however, are associated with certain limitations. In the first case, rather large amounts of the material are needed, since the minimum total surface area required for meaningful results lies in the range of 1 m^2^. For npAu exhibiting specific surface areas of 3–10 m^2^/g, for example, several 100 mg of the material are needed. Accordingly, such measurements were seldom done. In the second case, the electrochemically determined surface area in liquid phase does not necessarily reflect the one which is relevant for gas phase reactions. Ref. [53] summarizes results obtained for npAu with both techniques.

An attractive alternative for ordered and homogeneous pore systems, which spares regular experimental characterizations, was recently proposed in the literature [54]. It is based on the derivation of specific surface areas from structural parameters, specifically from the mean diameter *d*_*L*_ of the ligaments, constituting the pore system. As this novel approach allows for an elegant and unified analysis of the interplay of mass transport and catalysis in case of such materials, we briefly discuss the underlying considerations in the following.

The specific surface area *A*_*m*_ is defined as the ratio of the assessable surface area *A* and the mass *m* of a specimen and is related to *A*_*V*_, i.e., the surface area per volume *V* in the following way:

2 $$ A_{m} = A/m = A/V \cdot \rho_{p} = A_{V} /\rho_{p} $$

Here, *ρ*_*p*_ denotes the mass density of the porous material which is equivalent to the mass density *ρ* of the bulk material (in case of npAu: the mass density of Au) multiplied with the material filled fraction. This fraction equals to 1 minus the void fraction, which, in turn, is given by the porosity *ϕ*:

3 $$\rho_{p} = \left( {1 - \phi } \right) \cdot \rho$$

If pore systems are comprised of recurring structural motifs, they can be described as a periodic arrangement of identical elementary cells. Strictly speaking, this requirement, of course, is only fulfilled for crystalline porous materials, such as zeolites e.g., but principally also met by homogeneous self-similar structures, as given for npAu, where small partial volumes already reflect the pore system in total as far as pore diameters, pore shapes and the pore connectivity are concerned. In this case, the material can also be envisioned to consist of periodic cells exhibiting identical surface areas and the same specific surface area than the whole pore system. In other words, it is sufficient to consider such a unit cell to assess *A*_*m*_ and *A*_*V*_ instead of a macroscopic specimen [54].

To this end, let us assume that such cells are cubic and have an edge length *a*. In this case, their volume is given by *a*^*3*^. When furthermore assuming cylindrical ligaments making up the material-filled fraction within the cell, their surface area must be proportional to *r*_*L*_*⋅L*_*L*_ or *d*_*L*_*⋅L*_*L*,_ respectively, with *r*_*L*_ and *d*_*L*_ denoting their radius and diameter, respectively, and *L*_*L*_ their total length. As the ligaments of all cells need to be connected to jointly constitute the pore system of the material, *L*_*L*_ needs to be correlated with *a* (i.e.: *L*_*L*_ ~ *a*). In turn, for *A*_*V*_ the following proportionality can be derived:

4 $$A_{V} \sim \frac{{d_{L} \cdot L_{L} }}{{a^{3} }} \sim \frac{{d_{L} \cdot a}}{{a^{3} }} = d_{L} /a^{2} .$$

This expression can be simplified further: since the material-filled volume of an elementary cell is given by (1 − *ϕ*) *⋅ V* = (1 − *ϕ*) *⋅ a*^*3*^ and, at the same time, is proportional to the volume of the cylindrical ligaments i.e. *d*_*L*_^*2*^* ⋅ L*_*L*_ ~ *d*_*L*_^*2*^* ⋅ a*, it follows:

5 $$\left( {1 - \phi } \right) \cdot a^{3} \sim d_{L}^{2} \cdot a \Leftrightarrow a \sim 1/\sqrt {1 - \phi } \cdot d_{L}$$

meaning that, for a given porosity, *a* and *d*_*L*_ are actually related to each other. Accordingly, Eq. 4 can be rewritten as:

6 $$A_{V} \sim \frac{1 - \phi }{{d_{L} }} or: A_{V} = \zeta \cdot \frac{1 - \phi }{{d_{L} }}$$

showing that the (assumed) size of the unit cell *a* finally drops out of the equation. To convert the relation into an equation, a proportionality constant $\zeta$ was introduced in Eq. 6. Using furthermore Eqs. 2 and 3, for the specific surface area *A*_*m*_ the following formula finally results:

7 $$A_{m} = \frac{{A_{V} }}{{\rho_{P} }} = \frac{{\zeta \cdot \left( {1 - \phi } \right)}}{{d_{L} \cdot \rho_{P} }} = \frac{{\zeta \cdot \left( {1 - \phi } \right)}}{{d_{L} \cdot \left( {1 - \phi } \right) \cdot \rho }} = \frac{\zeta }{{d_{L} \cdot \rho }}\sim \frac{1}{{d_{L} }}$$

Accordingly, *A*_*m*_ is directly proportional to the inverse (mean) ligament diameter. The constant $\zeta$ is material specific and depends on the type of pore structure under consideration. Examples and associated values of $\zeta$ are given in ref. [54] and can range between ~ 0.5 and ~ 6.

**Fig. 3:**
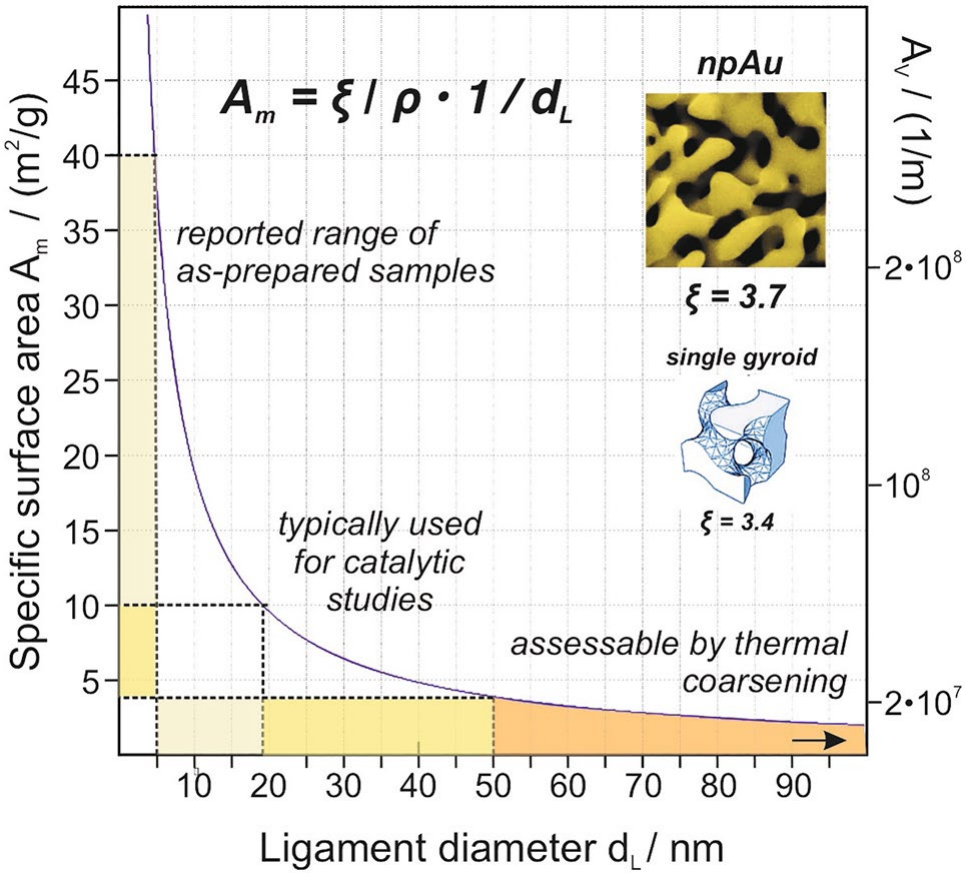
Dependence of the specific surface areas *A*_*m*_ and *A*_*V*_ on the mean ligament diameter, according to Eq. 7. For the majority of catalytic studies published in literature, npAu catalysts were used which exhibited ligament diameters between ~ 20 and 50 nm. For this size range surface areas between ~ 4 and 10 m^2^/g are expected. Larger ligament sizes can be obtained by subsequent thermal coarsening; in this case, however, the surface areas are typically too small for catalytic applications. The constant ${\upzeta }$ determined for npAu resembles that of a single gyroid structure, suggesting a certain comparability of the pore arrangements

In case of npAu, it can be taken advantage of the fact that ligament (*d*_*L*_) and the pore diameters (*d*_*P*_) are typically equal (see section above) so that also the latter can be used for the calculation instead of the former [38]. The constant $\zeta$ of npAu was determined on the basis of experimentally measured values for *A*_*m*_ by Detsi et al. Their obtained value of 3.7 indicates that npAu's porosity is similar to a so-called (single) gyroid pore structure (see, Fig. 3 and Ref. [54]). Figure 3 shows *A*_*m*_ as a function of the ligament diameter as derived from Eq. 7. The plot reveals that catalytically interesting surface areas between 4 and 10 m^2^/g are achievable for ligament sizes of npAu lying between 5 and 50 nm, i.e., for values which can be obtained with  established preparation protocols in a controllable fashion [55].

## Diffusion of Gases and Gas Mixtures in Porous Matter

As pointed out in the Introduction, the porosity of a heterogeneous catalyst does not only determine its specific surface area but also the mass transport within the material. Since the supply of reactants needs to take place via diffusion, which is restricted as compared to the bulk gas phase, the structural confinements posed by the porous system will play a role. These take effect in two different ways. On the one hand, collisions with the pore walls prevail over molecule–molecule interactions when the pore diameters fall below the mean free path of the molecules in the bulk phase. Around atmospheric pressure, this transition typically occurs in the mesoporous range and leads to Knudsen diffusion. On the other hand, the tortuous pore system only allows certain pathways for the diffusing gas molecules, resulting in longer distances to be overcome [2].

The first aspect can be dealt with comparatively easily. As the Knudsen diffusion coefficient only depends on the confining dimensions of the medium (and the molecular mass of the gas) and not on the gas environment (and its composition), it can be calculated based on kinetic gas theory [2]:

8 $$D_{K} = \sqrt {\frac{{8 \cdot d_{p}^{2} \cdot R \cdot T}}{{9 \cdot \pi \cdot {\text{M}}}} }$$

*T*: temperature, *M*: molecular weight of the gas, $d_{p}$: (mean) pore diameter.

In case molecular diffusion—characterized by the molecular diffusion coefficient *D*_*M*_—is also contributing, the resulting total diffusion coefficient *D*_*T*_, influenced by both mechanisms, is assessable by reverting to the so-called Bosanquet equation [2, 56]:

9 $$D_{T} = \left( {\frac{1}{{D_{M} }} + \frac{1}{{D_{K} }}} \right)^{ - 1}$$

**Fig. 4:**
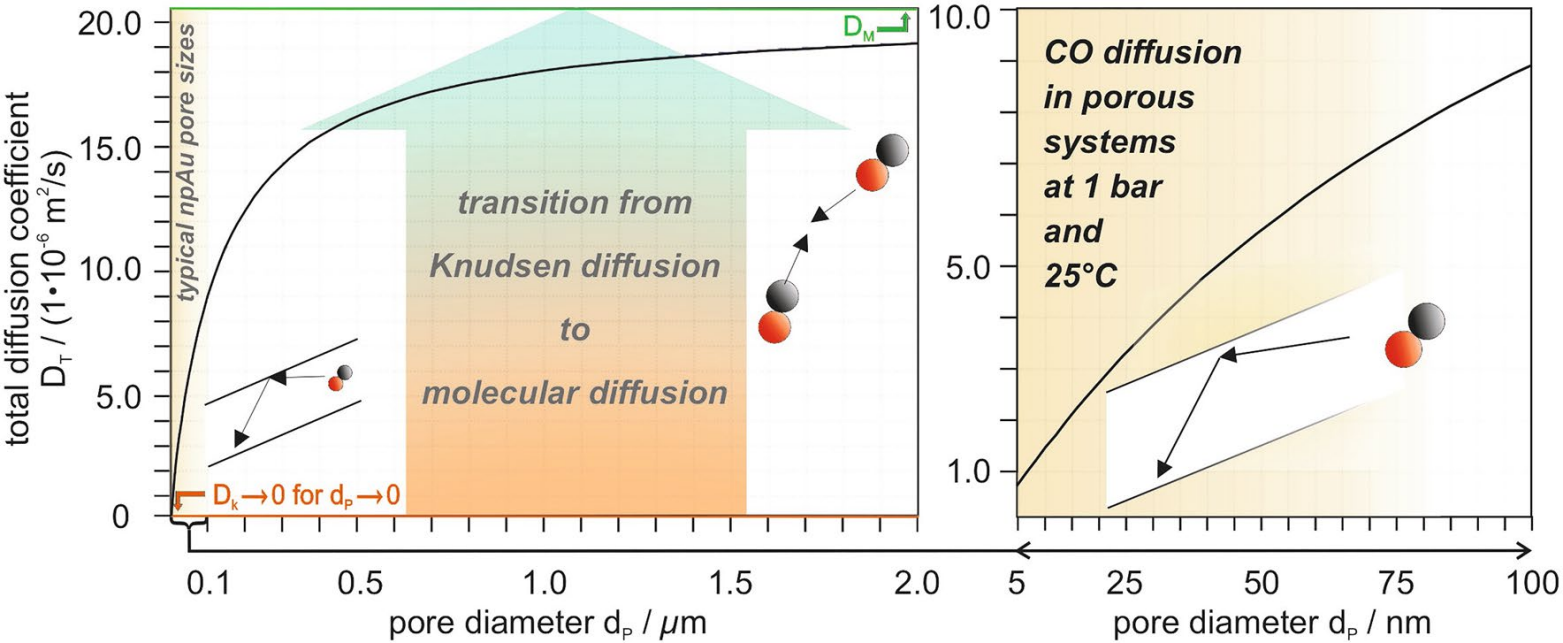
Dependence of the total diffusion coefficient *D*_*T*_ (Eq. 9) of CO on the pore diameter *d*_*p*_ when diffusing in a porous material at 1 bar and 25 °C. For large pore diameters (see left panel), *D*_*T*_ approaches *D*_*M*_, i.e., the molecular diffusion coefficient (20.4⋅10^–6^ m^2^/s, green line), determined by intermolecular collisions. For pore diameters being significantly smaller than the mean free pathway in the bulk gas phase (see right panel), *D*_*T*_ can be up to an order of magnitude smaller and is mostly determined by Knudsen diffusion. For 1 bar and 25 °C, this situation is given in case of the pore diameters typically occurring for as-prepared npAu samples

Figure 4 shows *D*_*T*_ of CO—taken as an example in view of the case study discussed in this article (low-temperature CO oxidation over npAu)—as a function of the pore diameter *d*_*p*_ at 25 °C and a pressure of 1 bar, i.e., for conditions under which most of the catalytic studies published in literature so far [43] were carried out. For pore sizes of 20–50 nm, representing typical values for npAu samples used in such experiments, the plot reveals that Knudsen rather than molecular diffusion dominates. Since the latter inversely depends on the pressure (according to simple kinetic gas theory), the molecular diffusion coefficient decreases at higher pressures. If it becomes significantly smaller than *D*_*K*_, *D*_*T*_ approaches *D*_*M*_. At 15 bar, for example, where the PFG NMR measurements discussed in the next section were performed, this is the case as *D*_*M*_ is only about half as large as *D*_*K*_.

Under catalytic reaction conditions, of course, not only one gas species but also other molecules are present in the gas phase. In contrast to *D*_*K*_*,* the resulting molecular diffusion coefficient *D*_*M,mix*_ is then dependent on the composition of the gas phase, according to [57]:

10 $$D_{M,mix,i} = \frac{{1 - \chi_{i} }}{{\mathop \sum \nolimits_{j \ne i} \frac{{\chi_{j} }}{{D_{M,i,j} }}}}$$

*χ*i: molar fraction of gas i

Here, *D*_*M,i,j*_ represent the binary diffusion coefficients which, in turn, can be calculated on the basis of the individual ones via [2]:

11 $$D_{M,i,j} = \left( {\frac{1}{{D_{M,i} }} + \frac{1}{{D_{M,j} }}} \right)^{ - 1}$$

Equations 10 and 11 predict that in the presence of a gas component in large excess the molecular diffusion coefficients of all other components will approach the value of this species, in particular if its molecular weight is distinctly smaller. Such a situation is often given in catalytic lab periments, where a carrier gas, such as He (see below) makes up the major part of the gas feed.

While for Knudsen diffusion basically only the (mean) pore diameter is decisive, more material-specific impediments result from the structural details of the pore system. To characterize this contribution, a quantity, called tortuosity *τ* was introduced in the literature as the factor by which diffusion of a gas molecule is slowed down as compared to straight pores [2]:

12 $$D_{E} = \frac{1}{{\uptau }} \cdot D_{T } \Leftrightarrow \tau = \frac{{D_{T} }}{{D_{E} }}$$

Accordingly, *τ* represents the ratio of *D*_*T*_—comprising already the influence of Knudsen diffusion— and the effective, i.e., actual diffusion coefficient *D*_*E*_ within the porous material. In this way, it is a means allowing to quantify the impact of the pore shapes, their spatial arrangement and their connectivity in an integral fashion. To this end, it is important that the diffusing molecules do not “stick” to the pore surfaces, meaning that molecule-pore wall interactions can essentially be described as hard-core repulsive interactions.

Aiming, however, at a more physical and more structure-specific interpretation, two factors slowing down the mass transport should be differentiated between: the effect of longer diffusive pathways within the porous material and the effect of pore size variations [58]. The importance of the latter aspect becomes apparent when taking into account that locations where the pore diameters become significantly smaller than their average may act as “bottle necks” for the diffusing molecules which are difficult to pass. A quantity introduced to consider this impact is the so-called constrictivity *δ* (0 < *δ* < 1 [59]), while contributions resulting from pathway elongations are subsumed in a separate number, unfortunately also called tortuosity in the literature, but denoted *τ’* in the following. On these grounds, the relation between *D*_*E*_ and *D*_*T*_ can alternatively expressed by [60, 61]:

13 $$D_{E} = \frac{\delta }{{\tau ^{{\prime}{2}} }} \cdot D_{T }$$

In line with this more physical approach, *τ’* enters Eq. 13 squared. It is easily comprehensible that a molecule can take different (porous) pathways, when diffusing along a pore and reaching a pore node. The larger the angle *θ,* by which the molecule is redirected when moving forward along one of them, the larger the additional diffusion length to be overcome as compared to a straight trajectory. Quantitatively, the elongation increases by *1/cos (θ)*. In addition, their statistical probability plays a role which also scales with *1/cos (θ)*, finally resulting in a 1/*cos*^*2*^* (θ)* ~ * τ’*^2^ dependence.

In contrast, the constrictivity bears on the diffusive flux only in a linear fashion. As shown by Peterson [59], *δ* depends on the ratio of the maximum and minimum pore cross section and can be estimated, using the following equation:

14 $$\delta \approx 1 - 0.21 \cdot \ln \left( {\frac{{A_{p,max} }}{{A_{p,min} }}} \right) = 1 - 0.21 \cdot \ln \left( {\frac{{d_{p,max}^{2} }}{{d_{p,min}^{2} }}} \right) = 1 - 0.42 \cdot \ln \left( {\frac{{d_{p,max} }}{{d_{p,min} }}} \right)$$

*A*_*p,max*_: maximum pore cross-sectional area, *A*_*p,min*_: minimum pore cross-sectional area, *d*_*p,max*_, *d*_*p,min*_: corresponding diameters

Due to the logarithmic dependence, the area ratio can be substituted by the ratio of the corresponding pore diameters or alternatively ligament diameters, when taking into account that the former and latter are typically identical for npAu. The analysis is simplified further by the invariance of the (Gaussian) ligament size distribution when normalizing it to the average ligament size *d*_*L*_ [39]. To assess *d*_*L,max*_*/d*_*L,min*_*,* its halfwidth, ranging from 0.5 to 1.5 of the mean value, may be used. On this basis, a value of 3 can be derived for the ratio for which, in turn, Eq. 14 yields a constrictivity of *δ*~ 0.5 for npAu.

Even though the differentiation between *δ* and *τ’* may be expedient to judge impediments for the mass transport along and perpendicular to the diffusive pathways, it is important to note that both quantities are not necessarily independent from each other. In case of low constrictivities, the probability of “bottle necks”, the molecules need to pass on their pathway through the pore system, statistically increases with the length, thus diminishing the share of longer pores to the diffusive flux. We will come back to this point later.

## Direct Measurement of Gas Diffusivities by PFG NMR

Over the last decades, PFG NMR has demonstrated an unprecedented potential to directly study the self-diffusion of molecules in porous materials [2, 18]. While in the liquid phase, such kind of studies are comparatively easy to conduct, measurements in the gas phase are distinctly more difficult due to the much lower (10^–3^) density of molecules, and only became possible with the advent of NMR instruments working at high magnetic field. The applicability of this technique was clearly demonstrated not only for measurements of self-diffusion in bulk gases, but also within porous matter, such as zeolites and oxides [2, 18]. In contrast, nanostructured metal materials have not been studied until very recently at the example of npAu [43], possibly because they have been considered not to be accessible by PFG-NMR. Specifically, the induction of eddy currents has to be expected for metallic specimen which can potentially lead to measurement artefacts.

Briefly, PFG-NMR is based on labelling and, after a controlled diffusion time *t*, unlabelling positions of diffusing molecules by applying magnetic field gradient pulses. For more details and an in-depth discussion, the interested reader is referred to the excellent book of J. Kärger et al. on this topic [2]. The property directly measured in PFG NMR is the mean square displacement (MSD), which is related to the self-diffusion coefficient and to the (known) diffusion time *t*, according to the Einstein relation for 3-dimensional diffusion:

15 $$<r^{2}>= 6 \cdot D \cdot t$$

* < r*^*2*^* >*: mean square displacement (MSD); *D*: self-diffusion coefficient.

The MSD and the corresponding self-diffusion coefficient are obtained from the attenuation *Ψ* of the PFG NMR signal, viz. the ratio of the PFG NMR signal magnitude with (*S*_*G*_) and without (*S*_*0*_) the field gradient pulses. In a typical PFG NMR experiment the attenuation is measured as a function of the magnetic field gradient amplitude (*g*), while keeping all other parameters fixed. Such dependencies of *Ψ* on *g* are referred to as PFG NMR attenuation curves. For normal, 3-dimensional diffusion, the following equation applies:

16 $${\Psi } = S_{G} /S_{0} = e^{{ - D \cdot q^{2} \cdot t}}$$

*q = γgδ*, where *γ* is the gyromagnetic ratio, and *δ* is the total effective gradient pulse duration for labelling or unlabelling gradient pulses.

Provided, however, that two ensembles of diffusing molecules exist, as given when, in the presence of a porous material, a part of the gas molecules diffuses inside and a part outside of the pore system, a bi-exponential decay behavior is expected:

17 $${\Psi } = S_{G} /S_{0} = p_{1} \cdot e^{{ - D_{1} \cdot q^{2} \cdot t}} + p_{2} \cdot e^{{ - D_{2} \cdot q^{2} \cdot t}}$$

Here, *p*_*1*_ and *D*_*1*_ denote the fraction and diffusion coefficient of the first ensemble (inside) and *p*_*2*_ and *D*_*2*_ the corresponding values of the second one (outside). The condition for being able to distinguish between both ensembles in the PFG NMR measurements is that the intra-particle MSD for a given diffusion time *t* is distinctly smaller than the physical dimension of a typical porous particle. Otherwise, the diffusing molecules can leave the pore system within the diffusion time and become part of the ensemble outside the material (or vice versa) so that both ensembles are mixed and become identical in the limit of large diffusion times, resulting in a mono-exponential decay.

**Fig. 5:**
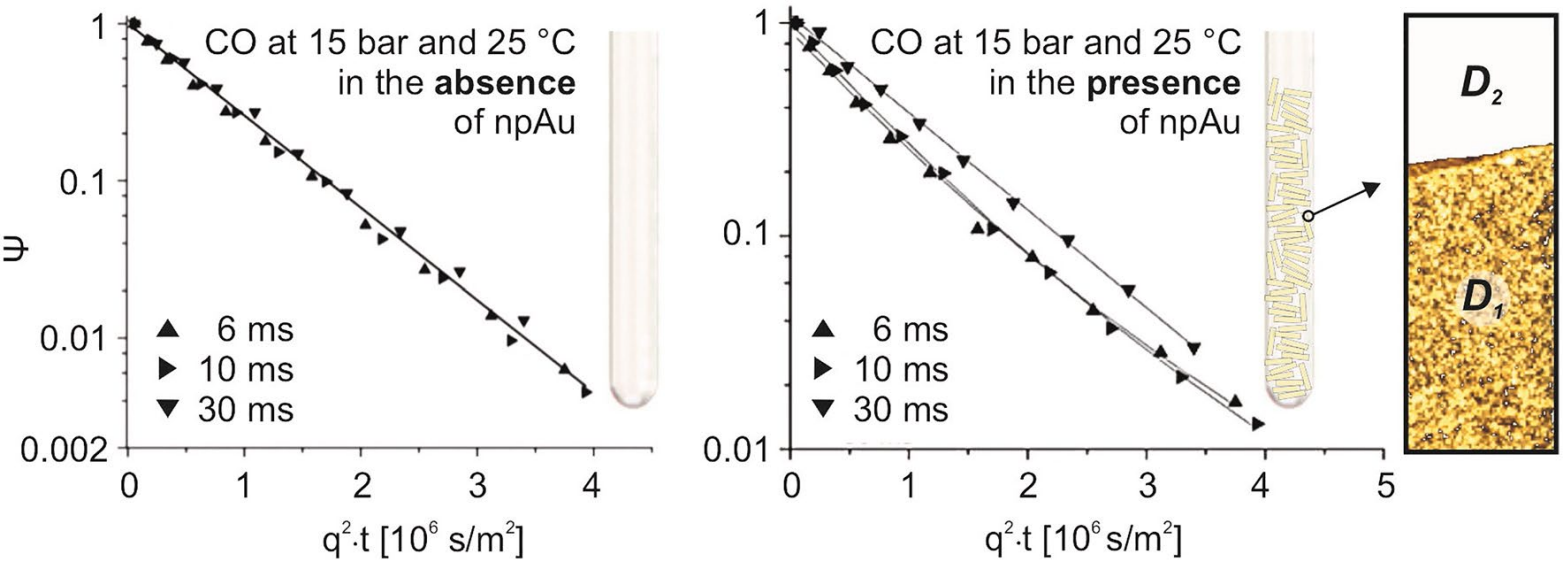
^13^C PFG NMR diffusion measurements for ^13^CO at 15 bar and 25 °C in the absence (bulk gas phase, left panel) and presence of npAu (right panel). In the latter case an NMR tube was filled with npAu discs (diameter: 3 mm, thickness: 200 microns). The signal attenuation *Ψ* was measured for 3 different diffusion times *t* in both cases: 6, 10 and 30 ms. In the presence of npAu, the curves show a biexponential behavior for 6 and 10 ms. By fitting both regimes, the diffusion coefficients *D*_*1*_ and *D*_*2*_ inside and outside npAu’s pore system could be determined (see Table 1)

Figure 5 and Table 1 summarize some of the results obtained for npAu and CO at 15 bar and room temperature. For diffusion times of 6 and 10 ms the measured PFG NMR attenuation curves clearly reveal deviations from a mono-exponential decay behavior as observed for the bulk gas phase (see Fig. 5, left panel). Rather, they can be fitted biexponentially according to Eq. 17, resulting in two distinctly different diffusion coefficients (Table 1). The larger one (*D*_*2*_) was found to match the value for the bulk gas phase (*D*_*bulk*_), hence belonging to CO molecules diffusing outside the pore system of npAu, whereas the smaller one (*D*_*1*_) can be assigned to the molecules diffusing inside the pore system. For t = 30 ms, however, where the mean square displacement already exceeds the smallest dimension of the npAu specimen studied (3 mm discs with a thickness of around 200 microns), only a mono-exponential decay can be discerned, indicating that this diffusion time is too long for distinguishing between both ensembles.

**Table 1:** Results of the least-square fitting of the data shown in Fig. 5, summarizing the diffusion coefficients of CO determined by PFG NMR at 15 bar and 25 °C for 3 different diffusion times *t* in the absence (*D*_*bulk*_) and presence of npAu (*D*_*1*_, *D*_*2*_)

*t* [ms]	*D*_*bulk*_ (10 ^−7^) [m^2^/s]	*RMSD*_*1*_ [µm]	*RMSD*_*2*_ [µm]	*D*_*1*_ (10 ^−7^) [m^2^/s]	*D*_*2*_ (10 ^−7^) [m^2^/s]	*D*_*2*_*/D*_*1*_
6	14 ± 1	149 ± 15	218 ± 15	7.0 ± 1.4	15 ± 2	2.1 ± 0.5
10	14 ± 1	201 ± 20	290 ± 20	7.2 ± 1.4	15 ± 2	2.1 ± 0.4
30	13 ± 1	430 ± 20	430 ± 20	10.5 ± 1	10.5 ± 1	–

In the presence of npAu and for diffusion times of 6 ms and 10 ms two gas ensembles can be distinguished between: one diffusing inside (subscript: 1) and one outside (subscript: 2) of npAu. In addition to the corresponding diffusion coefficients, the root mean square displacements (RMSD) are given. The last column shows the ratio *D*_*2*_/*D*_*1*_, corresponding to the tortuosity factor *τ*, according to Eq. 12

As already mentioned in the previous section, at 15 bar the contribution of Knudsen diffusion is minor and molecular diffusion governs the movement of CO inside the pores as it does in the surrounding bulk gas phase. Accordingly, this aspect cannot play a decisive role for *D*_*1*_ which is only half as large than *D*_*2*_, revealing that diffusion inside npAu is slowed down by a factor of 2 as compared to the bulk gas phase. Measurements with CH_4_, interacting very weakly with Au surfaces and included in the study for reference purposes, showed the same behavior. This similarity proves that the transient adsorption of CO on the surface of npAu—being a prerequisite for a catalytic conversion, of course—is not of importance either. Therefore, the decelerated mass transport can be fully ascribed to the structural impediments, subsumed in the tortuosity factor *τ*, which was introduced above. With *D*_*1*_ corresponding to *D*_*E*_ and *D*_*2*_ = *D*_*M*_ = *D*_*T*_, the ratio *D*_*2*_*/D*_*1*_ then directly reflects *τ* according to Eq. 12 (*τ* = *D*_*2*_*/D*_*1*_ = 2).

## Determination of Tortuosities by STEM Tomography

An alternative to assessing tortuosities of porous materials by PFG NMR diffusion measurements are microscopy-based methods. A technique which, in this respect, underwent a steep development in the recent years and proved its capability to provide such information and to furthermore give detailed insight into the mesoscopic structure of porous materials is STEM tomography [14–16]. To this end, thin electron transparent specimen in the range of a few 10 nm need to be prepared. These are then successively tilted with the sample holder in the microscope, covering typically an angle range between − 70° and 70°. For all tilting angles STEM images (around 100) are acquired, corresponding to 2D projections in the corresponding direction.

**Fig. 6:**
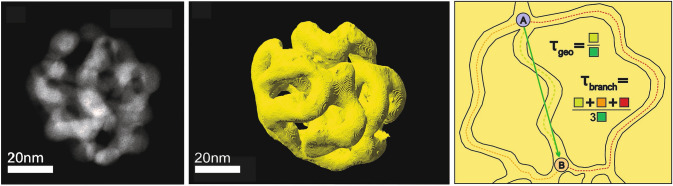
TEM micrograph of a npAu nanoparticle (left) and its 3D reconstruction (middle) based on STEM tomography. The picture on the right hand-side schematically illustrates the difference between the geometric and the branch tortuosity of a porous structure. (It shall be mentioned that *τ*_*geo*_ often is calculated not on the basis of curved pathways (such as the green dotted one) but on the basis of trajectories consisting of straight segments being somewhat shorter than the curved ones.)

Using appropriate algorithms [17, 62, 63], such a data set then allows for a 3D reconstruction of the specimen investigated. (For more details, the interested reader is referred to Refs. [14–16]). By applying a further numerical analysis, also tortuosity factors characterizing the pore system can be determined [64, 65]. Such methods, however, are based on specific geometric models [66]. The most straightforward one results in the so-called geometric tortuosity *τ*_*geo*_. It quantifies the ratio of the shortest average pathway for diffusing molecules, when moving from one point to another one within a given pore system, and the straight geometrical distance between these points (irrespective of the solid phase). If, alternatively, all possible pathways, the molecules may take within a structure of interconnected pores, are considered, the so-called branch tortuosity *τ*_*branch*_ is obtained, which is defined as the average length of this multitude as compared to the straight distance (see Fig. 6). (Accordingly, *τ*_*branch*_ is always larger than *τ*_*geo*_.)

In case of npAu, Mahr et al. recently published a STEM tomography study, using in this case nanoparticles, which were small enough for the measurements [67]. These can be prepared in analogy to macroscopic npAu samples (namely by dealloying AuAg nanoparticles instead [68]). Figure 6 shows the 3D reconstruction of such a sample, providing a clear visual impression of its pore system. The quantitative evaluation of the structure revealed a geometrical tortuosity of *τ*_*geo*_ = 1.2, whereas a branch tortuosity of *τ*_*branch*_ of 1.9 was derived. In addition to the statistical uncertainty associated with the tomographic data (20–25%), the limited volume and number of particles that can be studied in this way of course limits the numerical accuracy of such data further. Nevertheless, the insight into the structural details of a porous system provided by (S)TEM tomography can still be of substantial help to judge which of its characteristics predominately influence the diffusive transport.

When comparing the values obtained for *τ*_*geo*_ and *τ*_*branch*_ with *τ* derived from the PFG NMR measurements for npAu, a good agreement of the latter with *τ*_*branch*_ can be noticed at first sight. Yet, such an assessment does not take the influence of the constrictivity into account. As mentioned above, *δ* lies in the range of ~ 0.5 in case of npAu. On these grounds, a tortuosity factor *τ’* close to 1 results from Eq. 13, which rather reflects *τ*_*geo*_ than *τ*_*branch*_. This finding is in line with the consideration that low constrictivities lead to a preference for the shortest pathways through a pore system, i.e., lead to a decreasing contribution of longer alternatives to the diffusive transport. Hence, for npAu overall pore size variations seem to be more important than pathway elongations.

## Relationship Between Macro- and Micro-kinetics

The productivity of a catalyst depends on the interplay of its intrinsic activity, i.e., the rate of the underlying surface reaction, and its transport properties [11]. If the rate by which reactants are delivered to the inner, catalytically active surface areas (and/or by which products are transported back into the surrounding bulk gas phase) is significantly slower than the rate of the surface reaction, the former and not the latter determines the achievable conversion. The net effectiveness of a catalyst can be characterized by the so-called effectiveness factor *η*, which is defined as the ratio of the observed rate (*r*_*macro*_*)* and the rate of the surface reaction(s) (*r*_*micro*_) under steady state conditions:

18 $$\eta = \frac{{r_{macro} }}{{r_{micro} }}$$

This factor, representing a figure of merit for a given catalytic process, approaches 1 when transport limitations are negligible and decreases as such contributions become increasingly influential.

The macrokinetics, on the one hand, can be calculated in case of a continuous process on the basis of the conversion *X*_*i*_ which is observed at a pressure *p* and a temperature *T* for the reactant *i* added to the feed with an initial concentration $c_{i}^{0}$ (corresponding to the mole fraction *x*_*i*_):

19 $$r_{macro} = - \frac{1}{V}\frac{{dn_{i} }}{dt} = \frac{{\dot{V}}}{V} \cdot \left( {X_{i} \cdot c_{i}^{0} } \right) = \frac{{\dot{V}}}{V} \cdot X_{i} \cdot \frac{{x_{i} \cdot p}}{R \cdot T}$$

$\dot{V}$ here denotes the space velocity and *V* the reaction volume. In case of a porous catalyst, the latter equals the void volume *V*_*void*_ (non-material filled volume in the reactor, neglecting areas not filled with catalyst particles). The microkinetics *r*_*micro*_, on the other hand, is describable by the kinetic rate law which applies for the underlying catalytic surface reaction. For low-temperature CO oxidation over npAu (30 °C), which will be considered here, it is given by Eq. 1. For such a first order kinetics, *η* can be expressed in the following way [11]:

20 $$\eta = \frac{\tanh \left( \varphi \right)}{\varphi } \ with \ \varphi = \sqrt {\frac{{k_{V} \cdot \ell^{2} }}{{D_{E} }}}$$

Here, *φ* represents the Thiele modulus already alluded to in the Introduction. Apart from the microkinetic rate constant *k*_*V*_ and the effective diffusion coefficient *D*_*E*_ (of CO as the rate determining species in this case) within the porous catalyst (cf. Eqs. 12 or 13), it is dependent on a geometrical constant ($\ell$), representing a characteristic length of the catalyst particles practically employed. It is given by the ratio of their volume and their outer surface area and can be calculated for various geometries by the following general formula:

21 $$\ell = \frac{1}{{2 \cdot \left( {\frac{1}{x} + \frac{1}{y} + \frac{1}{z}} \right)}}$$

The quantities *x, y* and *z* here represent the lateral extensions of the catalyst particle along the three Cartesian coordinates. For spheres and cubes, for example, *x, y* and *z* are equal and correspond to the sphere diameter *d* or edge length *a*, respectively. For cylinders, *x* and* y* correspond to the diameter *d* while *z* to their height *h* .

As mentioned before, a central problem—when using the Thiele modulus formalism to determine achievable conversion rates on the basis of known microkinetics or to extract the microkinetics from observed macrokinetics—is missing knowledge about the effective diffusivity of the gases (*D*_*E*_) within the porous catalyst. While there is sufficient data in the literature regarding molecular diffusion coefficients in the bulk gas phase on the basis of which, in turn, total diffusion coefficients *D*_*T*_ (for pure gases and gas mixtures, see above) can be derived [2, 57, 69, 70], the material-related factors, i.e., tortuosities and constrictivities needed to determine *D*_*E*_ (Eqs. 12 or 13), are often unknown. In the literature, these were frequently guessed on the basis of assumptions or analogies to similarly structured pore systems. It can be easily shown for a random angle distribution of pore directions that $\tau^{\prime} = \sqrt 2$ (corresponding to the mean value of *1/cos*^*2*^*(θ),* see section 4.) results [71]. As demonstrated by theoretical studies on this topic [58, 72, 73], however, tortuosities can vary substantially for pore systems for which this condition is not (strictly) met. Accordingly, experimental values are inevitable when aiming at a quantitatively precise assessment of gas diffusivities in a porous catalyst material.

**Fig. 7:**
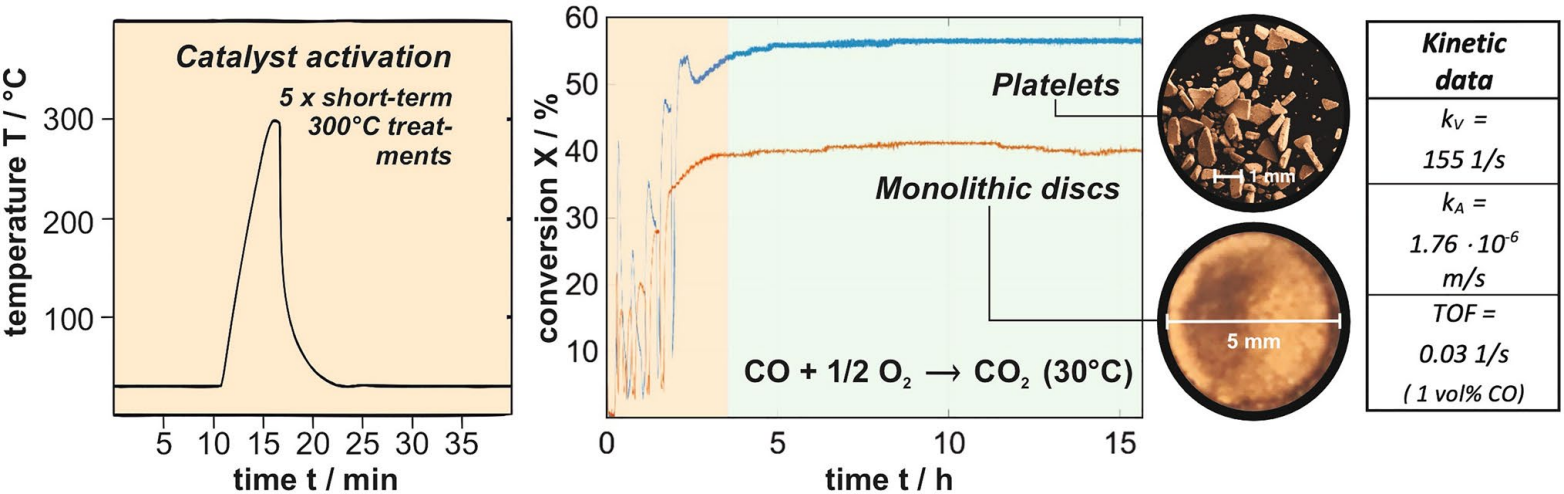
CO oxidation over npAu at 30 °C: Using a recently published activation procedure (left) [74]—consisting of several short 300 °C annealing steps—as-prepared npAu catalysts can be reliably activated for CO oxidation within a couple of hours. Monolithic npAu discs (diameter: 5 mm, thickness: 200 microns) activated in this way showed under the applied reaction conditions (p_Total_ = 1 bar, p(CO) = 10 mbar, p(O_2_) = 100 mbar, balanced with He as carrier gas) a conversion of ~ 40%. Taking advantage of the effective diffusion coefficient of CO determined by PFG NMR, the microkinetic data compiled on the right hand-side could be extracted. In addition to the monolithic samples, also npAu platelets (diameter: a few 100 microns, thickness: 200 microns) were investigated which were obtained by mechanical breakup of the 5 mm monoliths. In this way, the conversion could be increased to ∼60% (see next section)

In the following, we illustrate the possibilities given to disentangle macro- and microkinetics provided that such data are available. To this end, we revert to experiments carried out for low-temperature CO oxidation over npAu, summarized in Fig. 7. Using monolithic npAu discs with a thickness of 200 μm and a diameter of 5 mm (orange trace in the middle panel of Fig. 7), a steady state conversion of 40% (*X* = 0.4) could be achieved under the chosen reaction conditions (1 vol% CO, 20 vol% O_2_, carrier gas: He, 30 °C, atmospheric pressure, total gas flow: 40 sscm). It is worth noting that an activation procedure was applied which had been proven before to reproducibly lead to the same high conversion level for samples identically prepared by established dealloying protocols for free corrosion [74]. As depicted in the left panel of Fig. 7, it consists of several rapid heating steps to 300 °C (see left part of Fig. 7), which can be assumed to remove potential impurities on the surface (left, e.g., as remainders of the preparation) so that a defined and optimal surface state for the catalytic turnover can be reliably created.

To determine its kinetics, i.e., the microkinetics of the surface reaction, first the molecular diffusion coefficient (*D*_*M,mix*_) for CO under reaction conditions and its Knudsen diffusion (*D*_*K*_) coefficient for the given pore size (20 nm) need to be determined, utilizing Eqs. 8, 10 and 11. On basis of Eq. 8 (considering that, in the gas mixture, instead of *D*_*M*_* D*_*M,mix*_ enters the equation), then the total diffusion coefficient *D*_*T*_, comprising contributions of both diffusion mechanisms, can be calculated. Since at 1 bar the mean pore diameter is distinctly smaller than the mean free path of CO molecules in the bulk gas phase (see Fig. 4), *D*_*T*_ basically reflects *D*_*K*_. Notably, this circumstance reflects a situation, for which self-diffusion and transport diffusion become equivalent so that data for the former may be used even under reaction conditions. On contrary, in case of dominating molecular diffusion and noticeable local concentration gradients originating from the catalytic turnover at the pore walls, the corresponding coefficients will differ of course (unless a carrier gas, for example, is present in large excess ensuring a basically constant *D*_*M,mix*_ within the catalyst particle, i.e., a homogeneous molecular environment for the diffusing molecules).

One important difference between self- and transport-diffusion has to be taken into account in any event, however, when finally deriving *D*_*E*_ from *D*_*T*_ and tortuosities, determined by PFG NMR, for instance. When, in case of a working catalyst, reactants are consumed, the diffusive flux per *area* (according to the 1st Fick law) is relevant which can only take place in the void volume and not in the material-filled volume of the porous material. Accordingly, it is reduced by the porosity so that *D*_*E*_ not only scales with *1/ τ* (see Eqs. 12), but is also proportional to *ϕ* [60]:

22 $$D_{E} = \frac{\phi }{{\uptau }} \cdot D_{T}$$

In Table 2, all diffusion coefficients for CO relevant for the evaluation of the experiments in Fig. 7 are compiled [43].

**Table 2:** Overview of the relevant diffusion coefficients of CO needed to derive its effective diffusion coefficient D_E_ within npAu under the reaction conditions of Fig. 7

*D*_*M*_ [10^–6^ m^2^/s]	*D*_*M,mix*_ [10^–6^ m^2^/s]	*D*_*K*_ [10^–6^ m^2^/s]	*D*_*T*_ [10^–6^ m^2^/s]	*D*_*E*_ [10^–6^ m^2^/s]
1 bar 30 °C	1 vol% CO (10 vol% O_2_, 89 vol% He)	Pore diameter:* d*_*p*_ = 20 nm	$\left( {\frac{1}{{D_{M,mix} }} + \frac{1}{{D_{K} }}} \right)^{ - 1}$.	*τ* = 2.1 and *ϕ* = 0.7
20.4	83.4	3.2	3.1	1.0

While *D*_*M*_ represents the molecular diffusion coefficient measured by PFG NMR for the bulk gas and rescaled to 1 bar, *D*_*M,mix*_ refers to the gas mixture, used for the actual catalytic experiments. *D*_*K*_ is the Knudsen diffusion coefficient of CO in pores with a diameter of 20 nm, being typical for npAu. The total diffusion coefficient *D*_*T*_ taking contributions from molecular (in the reaction mixture) and Knudsen diffusion into account basically reflects *D*_*K*_. *D*_*E*_ results from *D*_*T*_ when considering also the tortuosity *τ* and porosity *ϕ* of npAu

To eventually determine the microkinetics, i.e., the rate constant *k*_*V*_ (Eq. 1), furthermore the characteristic length $\ell$ of the applied catalyst particles is needed. In case of the thin disc-shaped npAu monoliths used for the experiments presented in Fig. 7 (diameter *d*: 5 mm, thickness *h*: 200 μm), Eq. 21 predicts that $\ell$ approaches *h/2,* i.e., 100 μm.

Although Eq. 20 allows no analytical solution for *k*_*V*_, such an evaluation can be done numerically or graphically [43]. Based on the value for *k*_*V*_ obtained this way and given in Fig. 7, also the rate constant *k*_*A*_ (see Eq. 1) referred to the catalytic surface area is assessable. Taking advantage of Eq. 7, the following relationship can be derived:

23 $$k_{A} = k_{V} \cdot \frac{\phi }{1 - \phi } \cdot \frac{{d_{l} }}{c}$$

In addition, the turnover frequency *TOF* (number of catalytic revolutions per surface atom and second) may be obtained for the given reaction conditions (in case of the experiments shown in Fig. 7: *x*_*CO*_ = *0.01, p* = *1 bar, T* = *30 °C)* on these grounds:

24 $$TOF = N_{A} \cdot \frac{\sqrt 3 }{4} \cdot a_{Au}^{2} \cdot k_{A} \frac{{x_{co} \cdot p}}{R \cdot T}$$

Regarding the *TOF* as a measure often used to characterize the catalytic activity, two aspects have to be taken into account. First of all, and in contrast to *k* and *k*_*A*_, this quantity represents a rate and, as such, depends on the CO concentration or, as in Eq. 24, on *x*_*CO*_. Since it was found that the CO_2_ formation rate scales linearly with *x*_*CO*_ up to values of ~ 0.3 (30 vol%) [52], *TOFs* up to 1 s^−1^ can be achieved, when increasing the CO partial pressure in the feed. Secondly, a specific number density of surface atoms must be chosen as a basis for the calculation. In case of Eq. 24, a closed packed Au (111) was considered. Neither Au NPs nor npAu are expected to expose only (111) surfaces. Moreover, it is unlikely that all surface atoms contribute to the catalytic turnovers equally. Accordingly, the value, given in Fig. 7 for the *TOF*, represents only a lower limit. Nevertheless, the kinetic data compiled in the table on the right hand-side of Fig. 7 (*k*_*V*_*, k*_*A*_*, TOF*) represent the first results characterizing the genuine catalytic activity of npAu for low-temperature CO oxidation without ambiguities arising from unknown contributions of mass transport limitations [43].

## Optimization of a Catalysts’ Performance by Reducing Mass Transport Limitations

The CO oxidation experiments discussed above revealed a catalyst effectiveness *η* of about 70%, meaning that mass transport limitations played a non-negligible role, as reflected by a Thiele modulus $\varphi$ larger than 1 [43]. The latter has to be below ∼0.3 for *η* reaching values close to 1 [11].

Taking Eq. 20 into account, in principle different strategies are possible to decrease the Thiele modulus and thus to increase the catalytic productivity. On the one hand, pore diameters can be increased to reduce the contribution of Knudsen diffusion and thus to increase *D*_*E*_—an option principally existing for npAu by controlled thermal coarsening of the porosity. Yet, in this way also the specific surface inversely declines as inferable from Fig. 3. This loss must then be compensated by applying a larger amount of the catalyst. In cases where the catalyst material is expensive, as in case of npAu, such an option is unattractive. On the other hand, catalyst particles with a smaller characteristic length $\ell$ may be employed. Such an approach is often easier to realize and not connected with disadvantageous side effects. Other options, as reducing the tortuosity or optimizing the constrictivity of a porous catalyst, are typically beyond the realm of possibilities, since such parameters are material specific and cannot be changed readily.

**Fig. 8:**
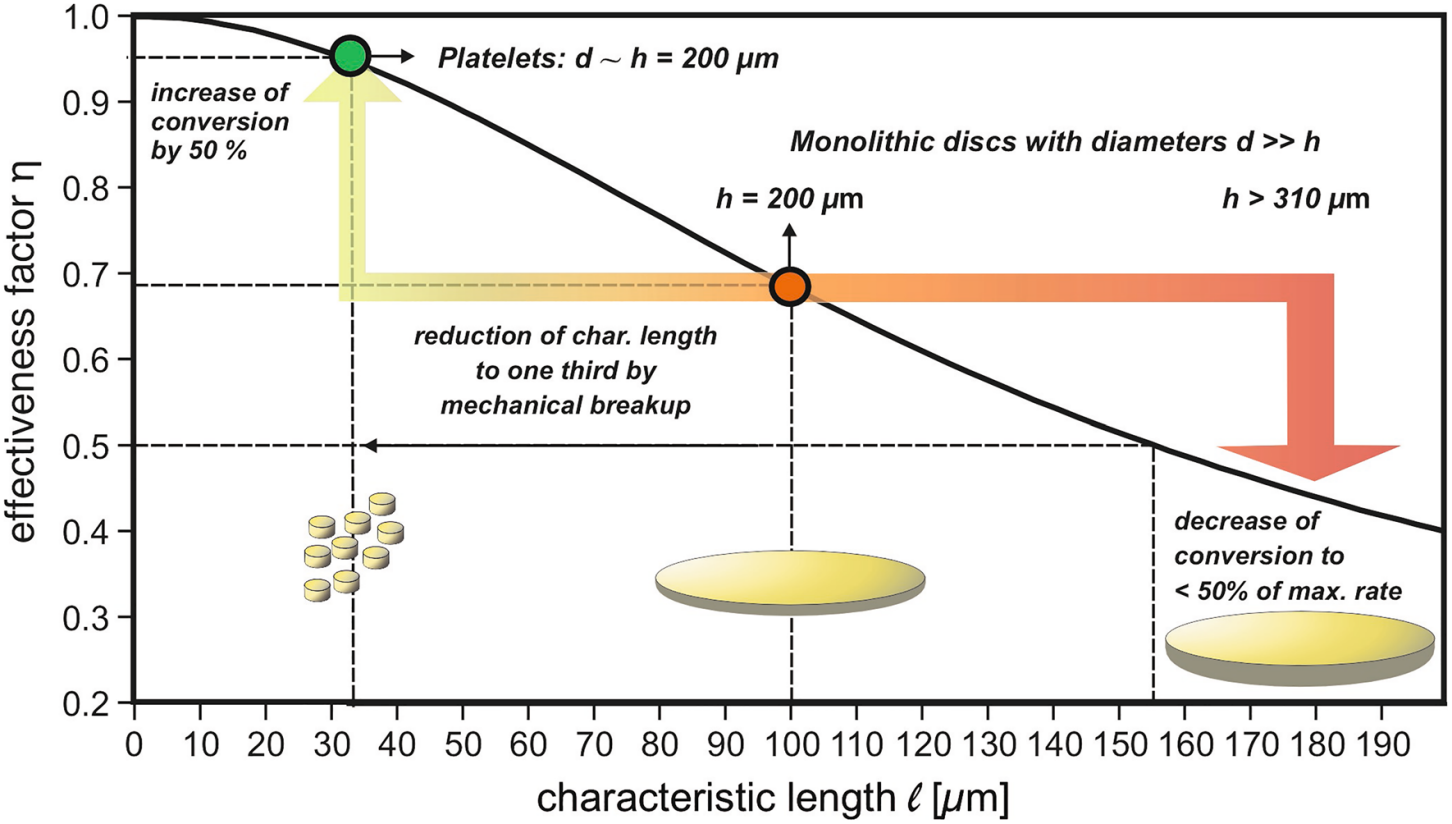
Effectiveness factor, corresponding to the ratio of observed rate (macrokinetics) and microkinetic rate, in case of CO oxidation over npAu at 30 °C as a function of the characteristic length of the catalyst particle actually used. When breaking up monolithic disc-shaped npAu samples exhibiting diameters (several mm), which are much larger than their thickness (a few 100 microns), – a sample type often employed in catalytic studies on npAu—into smaller platelets with diameters and thicknesses in the range of a few 200 microns, the conversion can be pushed to almost 100% of the maximum rate, reflecting the genuine catalytic potential of the material in the absence of mass transport limitations

Having knowledge about the microkinetics (i.e., *k*_*V*_) and the diffusive transport (i.e., *D*_*E*_) allows predicting how *η* varies with the characteristic length $\ell$ i.e., with the dimensions of the catalyst particles used. Figure 8 shows the results in case of low-temperature CO oxidation over npAu, indicating that catalytic yields close to the microkinetic limit (> 95%), can be achieved when $\ell$ is smaller than ∼30  μm (under the given experimental conditions). Taking into account that the data discussed so far were obtained with disc-shaped monolithic npAu samples exhibiting a characteristic length of 100 μm, it can be predicted that a reduction to a third of this value should lead to an increase of *η* from 70 to almost 100%. Reverting to Eq. 20, such a gain is expected when downsizing the disc diameters from the mm regime into the range of a few 100 μm. Experimentally, this can be realized by crushing the original npAu discs into smaller platelets. Applying a suitable procedure for this process (which does not deteriorate the fragile porous structure) provided conversions being 50% higher than those achieved with the larger discs, in accord with the predictions (see Fig. 7, blue trace) [43]. Figure 8 also reveals that for samples of the latter kind, the thickness sensitively influences the productivity. An increase by just 50%, i.e., to values above 300 μm, already shifts *η* into the range below 50%.

Overall, these results demonstrate the potential which is offered by techniques allowing to quantitatively characterize diffusion processes in porous catalysts. In combination with catalytic studies, novel perspectives emerge to quantitatively assess the degree to which catalytic processes are influenced by mass transport effects (under given reaction conditions) and indicate what measures must be taken to optimize conversions.

## Summary and Conclusions

The performance of a gas-phase porous heterogeneous catalyst depends on the kinetics of the underlying surface reactions and its surface area, on the one hand, and on the diffusion rates of the involved gas molecules in its pore system, on the other hand. It is the interplay of both aspects which finally determines the observed rates and achievable conversion levels. In this article, we aimed at elucidating new perspectives for quantitatively assessing and optimizing the overall process, resulting from the availability of novel catalytic materials with well-defined pore structures and the advent of elaborated experimental methods, allowing to characterize diffusive transport in porous matter.

Nanoporous gold, used in this article to illustrate these topics, can be considered as a good example in this respect. The fabrication of this novel porous metal catalyst by chemical or electrochemical dealloying is comparatively easy and leads to a homogeneous mesoporosity that can reproducibly be obtained. Its high catalytic potential for total as well as partial oxidation reactions at low temperatures does not rank behind (oxide) supported Au nanoparticles. While such traditionally prepared heterogeneous catalysts typically lack options to control porosity and surface properties, npAu illustrates the promising realm of novel strategies to synthesize catalytic materials with well-defined properties regarding catalytic turnover and mass transport. As a porous metal, npAu offers the additional advantage of a good thermal conductivity preventing the development of temperature gradients or hot spots under catalytic conditions.

In parallel, powerful experimental methods evolved in the last years, allowing to precisely characterize the transport properties. The seminal work of J. Kärger and coworkers demonstrated that, in particular, PFG NMR offers unrivaled possibilities in this respect. Taking advantage of the ongoing instrumental developments in this field, diffusivities of molecules in porous matter are measurable not only in the liquid but also in the gas phase. While oxides have been in the focus of research for a longer time, recent experiments with npAu indicated that also porous metals are assessable by this technique. On these grounds, the tortuosity *τ* of a pore system can be determined, which, in conjunction with knowledge about molecular and Knudsen diffusion coefficients of considered reaction gases, allows to get quantitative insight into their diffusivities being relevant for the mass transport therein. Besides, STEM tomography, as an additional or alternative characterization method, offers the opportunity to obtain local microscopic information about the structural details on the meso-scale. Such data can then help to evaluate which of them—pathway elongations or pore size variations—are mainly responsible for potential mass transport limitations, reducing the catalyst’s productivity.

On the basis of such quantitative knowledge about the transport properties of a porous catalyst, the underlying microkinetics, in terms of rate constants or TOFs, can be extracted from observed macrokinetics. For npAu, this approach led to kinetic data which, for the first time, enabled a reliable comparison with other gold catalysts regarding low-temperature CO oxidation—without ambiguities arising from unknown and interfering mass transport limitations. In this context, the spatially homogeneous and self-similar pore structure of npAu and the availability of a reliable activation protocol ensured that the data characterize the material and not just single samples. Under such circumstances, catalytic surface conditions are expected which do not vary locally and result in reproducibly achievable steady state conversions.

As illustrated at this example, in this way also the basis is laid for systematically improving the catalytic productivity. Here, different strategies are principally conceivable. One option, also existing for npAu to eliminate mass transport limitations, is given by increasing the (mean) pore diameter so that the contribution of Knudsen diffusion is reduced. Yet, it has to be taken into account in this context that also the specific surface area is likewise diminished. Alternatively, the tortuosity or constrictivity of the material may be optimized. As synthetic approaches for selectively varying these parameters do typically not exist, the most straightforward way is an adjustment of the catalytic particle size (i.e., their characteristic length). In case of CO oxidation over npAu, for example, a reduction of the dimensions of the npAu monoliths used led to an increase of the conversion close to the microkinetic limit, in line with the theoretical predictions.
